# Influence of person-organization fit on job satisfaction among pre-service preschool teachers of China: Mediation of teaching self-efficacy and perceived teacher competence

**DOI:** 10.1371/journal.pone.0351149

**Published:** 2026-06-08

**Authors:** Lu Xing, Xueqi Wu

**Affiliations:** 1 School of Preschool Education, Changsha Normal University, Changsha, China; 2 Faculty of Social Sciences Leisure Management, School of Education, Taylor’s University, Subang Jaya, Malaysia; 3 Faculty of Social Sciences Leisure Management, Taylor’s Culinary Institute, Taylor’s University, Subang Jaya, Malaysia; The Open University of Israel, ISRAEL

## Abstract

In China, a growing number of preschool education majors demonstrate reluctance to enter the profession upon graduation, posing a challenge to the sustainable development of the early childhood education workforce. Internships represent a critical period for pre-service teachers to develop their professional identity and capabilities. This study investigates the sustainability of pre-service teacher internships by examining the relationship between person-organization (P-O) fit and teachers’ job satisfaction, specifically focusing on the mediating roles of teaching self-efficacy and teaching competence. A quantitative survey was conducted with 493 pre-service preschool teachers (males 10.5%, females 89.5%) from Changsha Normal University between April and May 2024. Using Structural Equation Modeling (SEM), an integrated model of “values/needs matching → self-efficacy → competency development → teachers’ job satisfaction” was tested. The results indicate that P-O fit has a significant positive association with pre-service teachers’ job satisfaction, teaching self-efficacy, and teaching competence. Furthermore, teaching self-efficacy and teaching competence mediate the relationship between P-O fit and teachers’ job satisfaction, both independently and through a chain mediation effect. These findings suggest that to enhance the sustainability of internships and future retention, higher education institutions and kindergartens should prioritize value alignment and the progressive development of interns’ efficacy and competence.

## Introduction

China’s early childhood education (ECE) sector is currently witnessing a paradox of expansion and attrition. While policy initiatives over the past decade have successfully expanded the number of preschool education graduates [1], the industry struggles to convert these pre-service teachers into a stable workforce. Recent data indicates that despite a surge in qualified graduates, the growth of full-time preschool teachers has stagnated, with a notable decline observed in 2023 [2–5]. The high turnover rate of kindergarten teachers poses a severe challenge to early childhood education. According to the Chinese Society of Preschool Education Research [6], the average annual turnover rate for kindergarten teachers in China reached 28% in 2025. This structural instability incurs substantial organizational and developmental costs. Financially, the total cost of replacing a single teacher—encompassing direct expenditures for recruitment, onboarding, and training, alongside indirect losses from staff vacancies and potential enrollment attrition—is estimated at approximately 37,000 RMB. This replacement cost represents roughly 102.7% of a teacher’s average annual salary. Beyond the severe financial burden, the frequent disruption of the kindergarten’s micro-social environment profoundly impacts the children. As Tran and Winsler [7] demonstrated through large-scale longitudinal data, frequent teacher turnover is significantly negatively correlated with children’s cognitive, language, and social-emotional regulatory skills.

Empirical evidence consistently demonstrates that job satisfaction is one of the most consistent predictors of turnover intention and subsequent retention among teachers [8–10]. This “fleeing from the profession” phenomenon represents not only a waste of higher education resources but also a significant threat to the quality of early childhood care. Since the early turnover rate is high, pre-employment internships as the “litmus test” of a career, their experience quality directly determines whether students are willing to enter the profession. Therefore, targeting job satisfaction during the critical internship phase provides a proactive approach to addressing the broader retention crisis.

The educational internship serves as the critical “filter” in this career trajectory. It is the transitional phase where the idealized expectations of students collide with the realities of the kindergarten workplace—a phenomenon often described as “reality shock” [11]. Unlike in-service teachers who have adapted to the environment, interns are in a vulnerable state of professional identity formation. Evidence suggests that negative internship experiences are the primary predictor of pre-service teachers’ intention to abandon their teaching careers upon graduation [12]. Consequently, identifying the factors that bolster teachers’ job satisfaction during this specific developmental window is essential for improving retention rates.

In this study, we focus on Person-Organization (P-O) fit rather than Person-Job (P-J) fit as the primary antecedent. While P-J fit emphasizes the congruence between an individual’s abilities and the specific demands of a job (e.g., childcare and teaching tasks), P-O fit captures the alignment between individual and organizational values, encompassing value congruence and needs-supplies matching [13–15]. For pre-service kindergarten teachers, the internship is a vital period of organizational socialization. Research indicates that the high attrition rate and “reality shock” among early childhood educators are often driven more by incompatible organizational cultures, unsupportive management, and workplace climates than by the nature of the pedagogical work itself [16]. Even if pre-service teachers possess a high P-J fit, a perceived mismatch with the kindergarten’s specific values (low P-O fit) can significantly undermine their self-efficacy and lead to turnover intentions. Consequently, P-O fit serves as a more reliable indicator of the environmental interns navigate. While organizational psychology literature has extensively linked P-O fit to job satisfaction in corporate sectors [17], its application in the context of teaching internships is limited. However, a few recent studies have begun to adopt this model, demonstrating that higher P-O fit among early-career and preservice teachers significantly enhances their job satisfaction and organizational commitment while reducing burnout [18,19]. Despite these promising findings, the existing literature often treats the pathway from P-O fit to job satisfaction as a ‘black box’. We propose that for interns navigating a new environment, a high degree of P-O fit acts as a fundamental antecedent that reduces uncertainty and fosters a sense of belonging. However, merely “fitting in” contextually does not automatically guarantee sustained satisfaction; rather, this external compatibility must translate into internal psychological capacities. Therefore, the psychological mechanisms underlying this relationship remain a critical gap that needs to be explored.

This study aims to unpack the ‘black box’ of how organizational fit translates into job satisfaction by integrating Social Cognitive Theory. We posit that P-O fit is not just an emotional buffer but a facilitator of professional growth [20]. Specifically, we hypothesize a chain mediation process: a supportive organizational fit enhances Teaching Self-Efficacy (TSE) [21], which in turn promotes the acquisition of Perceived teacher competence (PTC). According to the Job Demands-Resources (JD-R) model [22], these personal resources (efficacy and competence) are crucial for combating burnout and achieving high job satisfaction [23].

Therefore, the present study utilizes Structural Equation Modeling (SEM) to test an integrated model: “P-O Fit → Teaching Self-Efficacy → Perceived Teacher Competence → Job Satisfaction.” By focusing on pre-service teachers in China, this research contributes to the international literature by clarifying the multi-pathway mechanism through which organizational environments shape early career trajectories. The findings offer empirical evidence for higher education institutions to redesign internship programs, shifting focus from mere placement to optimizing value alignment and self-efficacy.

### Theoretical background and hypotheses development

#### Person–Organization fit and teachers’ job satisfaction.

Person-Organization (P-O) fit theory posits that outcomes are optimized when there is compatibility between individuals and their work environments. This concept is rooted in Lewin’s field theory, which suggests that behavior is a function of the interaction between the person and the environment [24]. Contemporary research, particularly Kristof’s integrative framework, delineates P-O fit into two distinct dimensions: supplementary fit (value congruence between the individual and the organization) and complementary fit (needs-supplies matching) [14].

For pre-service teachers, the internship is a critical socialization phase. According to the Theory of Work Adjustment, job satisfaction arises when an organization’s reinforcers (e.g., culture, resources) meet the individual’s needs. While TWA holistically encompasses both satisfaction and actual retention, the current study is situated within the preservice internship period—a transitional phase prior to formal employment. Consequently, measuring actual long-term tenure is methodologically unfeasible in a cross-sectional design of interns. However, within the TWA framework, job satisfaction is conceptualized as the proximal psychological antecedent to tenure decisions. Empirical evidence consistently demonstrates that job satisfaction serves as a robust predictor of future retention and turnover intentions, particularly among educators [25,26]. Thus, this study focuses on job satisfaction as the critical theoretical endpoint and the most viable indicator of prospective professional retention during the internship stage.

When interns perceive a high level of value congruence with their kindergarten (e.g., shared educational philosophies), they experience reduced role ambiguity. Empirical studies indicate that P-O fit serves as a critical antecedent to satisfaction by fostering a sense of belonging and reducing turnover intention [17,27]. Recent research on Chinese preschool teachers specifically highlights that a lack of fit exacerbates burnout and diminishes job satisfaction [13]. A supportive P-O fit acts as a “protective factor,” buffering against the “reality shock” of the internship. Recent research indicates that value congruence specifically acts as a buffer against the high emotional labor demands in preschool settings [28]. Furthermore, empirical evidence suggests that when pre-service teachers perceive a misfit, their turnover intention crystallizes within the first month of internship, directly impacting their reported satisfaction [29].

Analytically, the mechanism linking P-O fit to job satisfaction lies in the reduction of cognitive dissonance and the fulfillment of psychological contracts. As demonstrated by Chen, Sparrow, and Cooper [30], when individuals’ values align with their organization, they experience higher interpersonal trust and expend significantly less emotional labor trying to conform to incongruent expectations. For pre-service teachers navigating the demanding emotional landscape of a kindergarten, this deep structural alignment generates a profound sense of psychological safety and authentic engagement, which intrinsically translates into sustained job satisfaction.

Hypothesis 1: Person-organization fit is positively associated with pre-service preschool teachers’ job satisfaction.

#### Person–Organization fit, teaching self-efficacy and perceived teacher competence.

Social Cognitive Theory (SCT) provides a robust framework for understanding how environmental factors influence personal agency [21]. Bandura identified “mastery experiences” and “social persuasion” as key sources of self-efficacy. We propose that a high P-O fit creates an optimal environment for these sources:

1. Resource Availability: A high needs-supplies fit implies the kindergarten provides necessary resources, enabling interns to accumulate mastery experiences [31].
2. Social Support: Value congruence often associated with better support from mentors, providing the social persuasion needed to bolster Teaching Self-Efficacy (TSE).

Regarding Perceived teacher competence (PTC), P-O fit acts as a contextual catalyst. Research by Keller et al. [32] suggests that when the environment satisfies teachers’ psychological needs (such as autonomy and relatedness), it frees up cognitive resources for skill acquisition and competence development. Conversely, a misfit may lead to psychological exhaustion, hindering the development of professional capabilities [13].

Fundamentally, a highly compatible organizational environment functions as an incubator for psychological safety [33]. When interns feel their educational values are respected and their developmental needs are met by the kindergarten’s resources, they are less likely to perceive critical feedback from mentors as a personal threat. Instead, this safety allows them to interpret mentorship as constructive social persuasion, thereby reinforcing their teaching self-efficacy [34]. Concurrently, by not expending excessive emotional energy on navigating organizational friction or value conflicts, interns can allocate their full cognitive capacity toward pedagogical skill acquisition, which directly accelerates the development of their perceived teacher competence.

Hypothesis 2: Person-organization fit is positively associated with pre-service preschool teachers’ teaching self-efficacy.

Hypothesis 3: Person-organization fit is positively associated with pre-service preschool teachers’ perceived teacher competence.

#### Teaching self-efficacy and teachers’ job satisfaction.

The relationship between self-efficacy and job satisfaction can be explained through the Job Demands-Resources (JD-R) model. In this model, self-efficacy is a crucial “personal resource” that helps individuals handle job demands and achieve work goals [23]. Pre-service teachers with high TSE act with greater assurance. They are more likely to view classroom challenges as manageable tasks rather than threats, a mindset that reduces stress and enhances satisfaction [35]. Studies have confirmed that teachers with high efficacy tend to form a positive cycle of “investment → success → satisfaction” [36].

From an analytical perspective, self-efficacy acts as an active cognitive buffer against workplace stressors [23]. Within the high-demand environment of early childhood education, interns inevitably encounter complex pedagogical challenges. High teaching self-efficacy alters the cognitive appraisal of these demands—transforming them from insurmountable threats into manageable challenges [37]. This proactive, problem-solving orientation mitigates emotional exhaustion and generates a continuous sense of mastery, which forms the psychological bedrock of job satisfaction.

Hypothesis 4: Teaching self-efficacy is positively associated with pre-service preschool teachers’ job satisfaction.

#### Teaching self-efficacy and perceived teacher competence.

While efficacy (belief) and competence (skill) are distinct constructs, they are recursively linked. Bandura’s axiom that “belief precedes action” suggests that TSE functions as a motivational mechanism [21]. Interns with high self-efficacy invest more effort and persist longer in the face of difficulties, which facilitates the transition from theoretical knowledge to practical Perceived teacher competence (PTC). The analytical driver of this relationship is behavioral persistence, a core component of Social Cognitive Theory [34]. Educational internships are inherently filled with trial and error. Self-efficacy provides the motivational resilience required to withstand initial pedagogical failures. Rather than withdrawing when facing classroom difficulties, highly efficacious interns are more likely to sustain their efforts. Instead of adopting avoidance strategies, they actively seek feedback and experiment with new pedagogical strategies. This continuous cycle of ‘action-reflection-modification’ is the practical pathway through which psychological belief is transmuted into perceived teacher competence [38]. This positive relationship has been substantiated by substantial empirical research. For instance, Holzberger, Philipp, and Kunter [39] demonstrated through a longitudinal analysis that higher levels of teacher self-efficacy significantly predict the development of instructional quality. Furthermore, a comprehensive meta-analysis by Klassen and Tze [40] confirmed a direct and significant empirical link between teachers’ self-efficacy beliefs and their overall teaching effectiveness and competence.

Hypothesis 5: Teaching self-efficacy is positively associated with pre-service preschool teachers’ perceived teacher competence.

#### Perceived teacher competence and teachers’ job satisfaction.

Competence is a fundamental factor in professional well-being. When pre-service teachers perceive themselves as competent—able to effectively manage a class and foster child development—they experience a sense of accomplishment. Research by Wang G. et al. [41] indicates that preschool teachers’ competence positively predicts occupational well-being by mitigating the negative effects of stress. Similarly, Yin et al. [42] found that competence enhances satisfaction through self-efficacy and professional identity. Therefore, as interns transition from novices to proficient practitioners, the mastery of core skills (e.g., child care and education) directly contributes to a more satisfying internship experience.

Analytically, this relationship is rooted in the fulfillment of the basic psychological need for competence, a core tenet of Self-Determination Theory [43]. As pre-service teachers successfully execute complex tasks—such as designing effective activities or soothing distressed children—they transition conceptually from peripheral learners to core contributors within the kindergarten. This tangible validation of their professional capabilities fulfills their intrinsic need for achievement and autonomy, thereby directly elevating their overall job satisfaction.

Hypothesis 6: Perceived teacher competence is positively associated with pre-service preschool teachers’ job satisfaction.

#### The mediating role of teaching self-efficacy and perceived teacher competence.

Integrating the above theoretical perspectives, we propose a multi-step mechanism. P-O fit should not be viewed merely as a static condition but as an antecedent that activates a psychological growth process.

1. Independent Mediation: P-O fit enhances job satisfaction indirectly by separately boosting self-efficacy (psychological capital) and competence (human capital). Keller et al. [32] found that fit stimulates competence development by enriching self-efficacy.
2. Chain Mediation: There is a sequential pathway. A supportive environment (P-O Fit) fosters the belief in one’s ability (TSE), which drives the effort required to master skills (PTC), ultimately leading to higher teachers’ job satisfaction (TJS). This aligns with findings that teachers with high self-determination and efficacy achieve better psychological well-being [44].

Empirical studies on pre-service teachers affirm this sequential ‘belief-action’ mechanism. For instance, recent research indicates that self-efficacy acts as a self-regulatory element that explicitly ‘activates’ professional competencies; teachers who believe in their capacity are more likely to persist in challenging situations, thereby acquiring actual skills [45]. Conversely, low efficacy has been linked to higher expectations of ‘reality shock’ and a failure to develop necessary coping strategies during the induction year. Thus, we argue that P-O fit first secures the psychological belief (efficacy), which then enables the behavioral acquisition of competence [46].

Analytically, this chain mediation represents a sequential internalization of resources. Drawing upon the Conservation of Resources (COR) theory [47], a favorable external environment (high P-O fit) provides the initial resource reservoir. However, interns do not translate this environmental advantage directly into complex pedagogical skills. Rather, the external support is first internalized as a vital psychological resource (teaching self-efficacy). This robust psychological foundation subsequently fuels the behavioral effort required to acquire human capital (perceived teacher competence). Ultimately, the successful accumulation of both psychological and human capital within a supportive environment generates the highest levels of job satisfaction. This sequential “environment → belief → action → affect” pathway analytically justifies the multi-step mediation mechanism.

Hypothesis 7a: Teaching self-efficacy mediates the relationship between P-O fit and teachers’ job satisfaction.

Hypothesis 7b: Perceived teacher competence mediates the relationship between P-O fit and teachers’ job satisfaction.

Hypothesis 7c: Teaching self-efficacy and Perceived teacher competence play a chain mediating role in the relationship between P-O fit and teachers’ job satisfaction.

**Fig 1:**
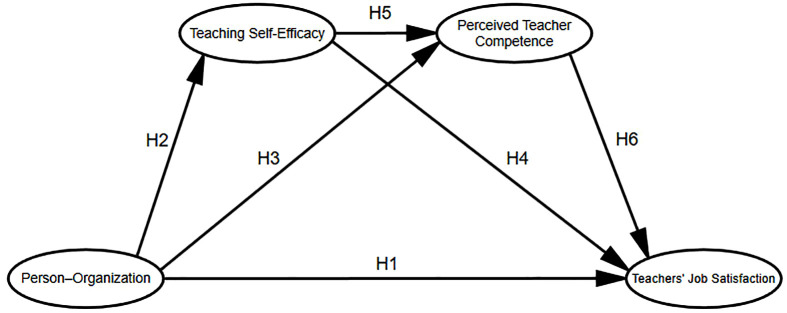
Model of person-organization-fit and teaching self-efficacy and teachers job contentment.

This research suggested a chain mediation model (Figure 1).

## Materials and methods

### Participants and procedure

This study employed a cross-sectional survey design. Participants were recruited using purposive sampling from pre-service preschool education students at Changsha Normal University in Hunan Province, China. The data collection period spanned from April 3 to May 21, 2024. The inclusion criteria were: (1) current enrollment as an undergraduate student majoring in preschool education; (2) completion of at least one educational internship lasting more than four weeks.

Written informed consent was obtained electronically from all participants. Before starting the survey, participants were presented with a detailed explanation of the study’s purpose, their rights, and the confidentiality of their data. They were required to click a button labeled ‘I agree to participate’ to confirm their consent and proceed to the questionnaire. Participants could withdraw at any time by closing the browser. As the study targeted undergraduate students, all participants were over 18 years of age, and no minors were involved.

The survey was administered via an online platform (powered by www.wjx.cn). The questionnaire was anonymous, and participants were assured that their responses would be used solely for academic research purposes. A total of 557 questionnaires were initially collected. Data cleaning was conducted following the guidelines of Hair et al. [48]. We excluded 64 invalid questionnaires based on the following criteria: (1) incomplete responses (n = 38); (2) response time being significantly shorter than the reasonable threshold (less than 120 seconds); and (3) “straight-lining” patterns (selecting the same option for all items) (n = 26). Consequently, 493 valid responses were retained for the final analysis, yielding an effective response rate of 88.51%. The final sample consisted of 52 males (10.5%) and 441 females (89.5%), aged between 18 and 22 years (M = 20.4, SD = 1.2), which is representative of the gender distribution in China’s preschool education sector.

The study was conducted in accordance with the Declaration of Helsinki. The protocol was reviewed and approved by the Ethics Committee of Changsha Normal University (Protocol code:2024–59).

### Measures

All instruments were adapted from established scales with proven reliability and validity. Through discussions with experts and scholars, we modified and optimized some scales to eliminate problems that were not consistent with the topic of this research, with these measures used to adapt to the current research background (Table 1).

**Table 1:** Measurement model, descriptive statistics.

Constructs	Unstd.	SE	T-Value	P	Std.	SMC	CR	AVE	Cronbach’s alpha
*Person–Organization (PO)* [17,49]							0.877	0.588	0.876
My personal values are very similar to those of my kindergarten.	1				0.743	0.552			
My values and traits can be reflected in the internship at the kindergarten.	0.942	0.057	16.593	***	0.78	0.608			
The material and spiritual resources provided by kindergarten work are very consistent with the job I want to find.	0.977	0.059	16.632	***	0.782	0.612			
The work of a kindergarten teacher is very much in line with my aspirations.	0.965	0.059	16.392	***	0.77	0.593			
The education and training I received are consistent with the needs of working in a kindergarten.	0.981	0.061	16.135	***	0.758	0.575			
*Teaching Self-Efficacy (TSE)* [50,51]							0.927	0.679	0.927
When children have difficulty understanding, I can provide some explanations of examples to help them understand.	1				0.87	0.757			
I am able to use a variety of teaching methods to design activities that are lively and interesting.	0.969	0.039	24.606	***	0.846	0.716			
I can easily handle the ‘disruptive’ behavior of some children during activities.	0.929	0.038	24.293	***	0.84	0.706			
I can stimulate and mobilize children’s enthusiasm to participate in activities.	0.953	0.04	23.91	***	0.832	0.692			
During the activities, I can give children the opportunity to fully express and make decisions.	0.917	0.043	21.251	***	0.776	0.602			
I can stimulate children’s creativity and cultivate children’s creative thinking.	0.88	0.041	21.251	***	0.776	0.602			
*Perceived Teacher Competence (PTC)* [52]							0.949	0.698	0.948
I have mastered the basic methods of scientifically caring for young children in their daily lives.	1				0.77	0.593			
I am familiar with the characteristics and laws of children’s physical and mental development.	1.011	0.049	20.472	***	0.839	0.704			
I am able to assist the childcare staff in carrying out routine childcare and sanitation work in the class.	0.985	0.05	19.512	***	0.808	0.653			
I can design educational activity plans based on children’s interests, needs, and age characteristics.	1.023	0.048	21.234	***	0.864	0.746			
I am able to flexibly use a variety of methods to implement educational activities.	1.001	0.049	20.535	***	0.841	0.707			
I can effectively observe children’s performance in activities and provide appropriate guidance when children need it.	1.074	0.048	22.23	***	0.895	0.801			
I can use observation, interviews, home-based cooperation and other methods to understand and evaluate young children.	0.977	0.047	20.783	***	0.849	0.721			
I can use evaluation results to analyze and improve educational activities.	0.946	0.048	19.682	***	0.813	0.661			
*Teachers’ Job Satisfaction (TJS)* [53,54]							0.887	0.612	0.887
I am contented with the teaching conditions of the kindergarten.	1				0.76	0.578			
I am contented with the director’s management style.	0.995	0.058	17.129	***	0.773	0.598			
My efforts can be recognized by others.	0.983	0.059	16.652	***	0.753	0.567			
I find teaching interesting, and I am happy being around children.	1.01	0.057	17.613	***	0.793	0.629			
My efforts receive positive responses from the children, and I enjoy the process.	1.019	0.055	18.466	***	0.83	0.689			

**Note:** SE Standard Error, P p-value, SMC Squared Multiple Correlations, CR Composition reliability, AVE Average variance extracted. ***p < 0.001. PO = Person-Organization Fit; TSE = Teaching Self-Efficacy; PTC = Perceived teacher competence; TJS = Teachers’ Job Satisfaction.

Minor modifications were made based on pilot feedback from three experts in teacher education to enhance content validity. All items were rated on a 7-point Likert scale ranging from 1 (completely disagree) to 7 (completely agree). This study’s data was collected in Chinese. For accuracy, a Chinese-English parallel version is provided in the appendix.

1. Person-Organization Fit (P-O Fit): This construct was measured using a 5-item scale adapted from Cable and Wang [17,49]. It assesses both value congruence and needs-supplies fit.
2. Teaching Self-Efficacy (TSE): We adapted 6 items from Bandura’s guidelines and previous studies [50,51] to measure interns’ beliefs in their instructional capabilities.
3. Perceived Teacher Competence (PTC): This variable was assessed using an 8-item scale [52] focusing on practical skills, such as daily care routines, activity design, and child assessment.
4. Teachers’ Job Satisfaction (TJS): A 5-item scale derived from relevant literature [53,54] was used to measure satisfaction with the internship environment, management, and sense of achievement.

### Data analysis

Data analysis was performed using SPSS 26.0 and AMOS 26.0. We adopted a two-step structural equation modeling (SEM) approach [55] to test the hypotheses.

#### Measurement model assessment.

Confirmatory Factor Analysis (CFA) was first conducted to evaluate the reliability and validity of the measurement model. Internal consistency was assessed using Cronbach’s alpha (> 0.70) and Composite Reliability (CR > 0.70). Convergent validity was evaluated using the Average Variance Extracted (AVE > 0.50) and standardized factor loadings (> 0.50) [48,56]. Discriminant validity was examined using the Fornell-Larcker criterion, which requires the square root of the AVE for each construct to exceed its correlations with other constructs [57].

#### Structural model assessment.

The fit of the structural model was evaluated using the Maximum Likelihood (ML) estimation method. Model fit was assessed based on standard indices: Chi-square/df ratio (< 3.0), CFI (> 0.90), TLI/NFI (> 0.90), RMSEA (< 0.08), and SRMR (< 0.08) [58,59].

#### Mediation analysis.

To test the mediating effects (indirect paths), we utilized the bootstrapping method with 5,000 resamples. The 95% bias-corrected confidence intervals (CI) were calculated. An indirect effect is considered statistically significant if the 95% CI does not contain zero [60,61].

## Results

### Measurement model assessment

To strictly monitor common method variance (CMV), we employed the Common Latent Factor (CLF) method in AMOS since the Harman’s single-factor test showed a borderline result (52.19%). We compared the standardized regression weights of the model with and without a common latent factor. The differences in regression weights were found to be minimal (all Δ < 0.200), indicating that common method bias did not significantly distort the structural relationships in this study. We have also adopted comparison between the CFA single-factor model and the theoretical model. The results show that the fit of the single-factor model (χ²/df = 8.781, RMSEA = 0.126, CFI = 0.780) is much worse than that of the theoretical model (χ²/df = 1.712, RMSEA = 0.038, CFI = 0.980). Therefore, the results indicate that CMV has no significant effect, the data meets the requirements and can continue to be used. This step ensures the validity of the subsequent CFA and structural models.

Before testing the structural relationships, a Confirmatory Factor Analysis (CFA) was conducted using the Maximum Likelihood method [62] to assess the reliability and validity of the constructs (P-O Fit, Teaching Self-Efficacy, Perceived Teacher Competence, and Teachers’ Job Satisfaction).

As shown in Table (Table 1), the factor loadings for all items ranged from 0.743 to 0.895, exceeding the recommended threshold of 0.501. The Cronbach’s alpha and Composite Reliability (CR) values for all constructs were greater than 0.80, well above the 0.70 benchmark, indicating high internal consistency. Furthermore, the Average Variance Extracted (AVE) values ranged from 0.588 to 0.698, exceeding the 0.50 threshold, which supports adequate convergent validity.

Discriminant validity was assessed using the Fornell-Larcker criterion [57]. As presented in Table 2, the square root of the AVE for each construct (shown in bold on the diagonal) was greater than its highest correlation with any other construct. This confirms that the constructs are empirically distinct.

**Table 2:** Analysis of discriminant validity [57].

Constructs	Mean	SD	PTC	TSE	TJS	PO
**PTC**	4.797	.988	**.835**			
**TSE**	4.631	.979	.795	**.824**		
**TJS**	4.591	.991	.699	.729	**.782**	
**PO**	4.026	1.230	.613	.587	.630	**.767**

**Note:** Correlations among constructs are represented by off-diagonal values. Values in bold are the square roots of AVE. SD = Standard deviation; PO = Person-Organization Fit; TSE = Teaching Self-Efficacy; PTC = Perceived teacher competence; TJS = Teachers’ Job Satisfaction.

### Structural model test

The structural model was evaluated using Maximum Likelihood (ML) estimation. The fit indices indicated that the hypothesized model fits the data well (Table 3): χ2 = 421.142, df = 246, χ2/df = 1.712(<3.0) CFI = 0.980 (>0.90), NFI = 0.954 (>0.90), RMSEA = 0.038 (<0.08), and SRMR = 0.035(<0.08). These indices suggest the theoretical model aligns well with the observed data.

**Table 3:** Model fitting index.

Fit indices	χ2	DF	χ2/DF	GFI	RMSEA	CFI	NFI	AGFI	SRMR
Reference value	–	–	<3	>0.8	<0.08	>0.9	>0.9	>0.9	<0.08
Model value	421.142	246.000	1.712	.932	.038	.980	.954	.918	.035

**Note:** χ2 Chi-Square Minimum Fit Function, DF Degrees of Freedom, χ2/DF Chi-Square to Degrees of Freedom ratio, GFI Goodness of Fit Index, AGFI Adjusted Goodness of Fit Index, CFI Comparative Fit Index, RMSEA Root Mean Square Error of Approximation, NFI Normed Fit Index, SRMR Standardized Root Mean Square Residual.

### Hypothesis testing (direct effects)

The standardized path coefficients (β) and significance levels for the direct effects are summarized in Table 4.

**Table 4:** Hypothesizes testing.

Hypotheses	Constructs	Unstandardized path coefficient	SE	C.R.	P	Standardized path coefficient	Results
Model with mediators							
H2	PO → TSE	.503	.056	8.982	***	.587	Supported
H5	TSE → PTC	.643	.065	9.892	***	.664	Supported
H3	PO → PTC	.185	.054	3.426	***	.223	Supported
H1	PO → TJS	.208	.045	4.622	***	.259	Supported
H4	TSE → TJS	.376	.068	5.529	***	.401	Supported
H6	PTC → TJS	.214	.074	2.892	***	.221	Supported
Model without mediators							
H1	PO → TJS	.500	.044	11.298	***	.631	Supported

**Note:** ***p < 0.001. PO = Person-Organization Fit; TSE = Teaching Self-Efficacy; PTC = Perceived teacher competence; TJS = Teachers’ Job Satisfaction.

First, Person-Organization (P-O) Fit had a significant positive predictive power on Teaching Self-Efficacy (β = 0.587, p < .001) and Perceived teacher competence (β = 0.223, p < .001), supporting H2 and H3.

Second, Teaching Self-Efficacy positively predicted both Perceived teacher competence (β = 0.664, p < .001) and Teachers’ Job Satisfaction (β = 0.401, p < .001), supporting H5 and H4.

Third, Perceived teacher competence was positively associated with Teachers’ Job Satisfaction (β = 0.221, p < .001), supporting H6.

Finally, P-O Fit maintained a significant direct effect on Teachers’ Job Satisfaction (β = 0.259, p < .001) even after accounting for the mediators, supporting H1.

Collectively, these results confirm that P-O Fit not only directly influences satisfaction but also initiates a pathway of professional development.

### Mediation analysis (indirect effects)

To rigorously test the mediating roles of Teaching Self-Efficacy (TSE) and Perceived Teacher Competence (PTC), we employed the bootstrapping method with 5,000 resamples and 95% bias-corrected confidence intervals (CI). The results of the mediation analysis are presented in Table 5.

**Table 5:** Mediation analysis.

SIE	Point Estimate	Product of Coefficients		Bootstrapping = 5000				Ratio %
				Bias-Corrected 95%CI		Percentile 95%CI		
		SE	Z	Lower	Upper	Lower	Upper	
Indirect Effect								
PO-TSE-PTC-TJS	.069	.027	2.556	.025	.136	.021	.131	13.64%
PO-TSE-TJS	.189	.040	4.725	.123	.280	.120	.276	37.35%
PO-PTC-TJS	.040	.017	2.353	.012	.083	.009	.077	7.91%
Total	.298	.041	7.268	.227	.386	.225	.383	58.89%
Direct Effect								
PO-TJS	.208	.045	4.622	.124	.300	.124	.299	41.11%
Total Effect								
PO-TJS	.506	.054	9.370	.410	.620	.410	.620	100.00%
Model without mediators								
PO-TJS	.500	.044	11.364					

**Note:** PO = Person-Organization Fit; TSE = Teaching Self-Efficacy; PTC = Perceived teacher competence; TJS = Teachers’ Job Satisfaction.

#### Indirect effect via self-efficacy (H7a).

The indirect path from P-O Fit to Teachers’ Job Satisfaction via TSE was significant (β = 0.189, p < .001). The 95% CI [0.123, 0.280] did not include zero, confirming the mediating role of self-efficacy. This path accounted for 37.35% of the total effect.

#### Indirect effect via competence (H7b).

The indirect path via PTC was also significant (β = 0.040, p < .001, 95% CI [0.012, 0.083]). However, the effect size was relatively smaller (7.91% of the total effect), suggesting that competence acts as a partial mediator.

#### Chain mediation effect (H7c).

The sequential mediation path (P-O fit → TSE → PTC → TJS) was significant (β = 0.069, p < .001). The 95%CI [0.025, 0.136] excluded zero. This finding supports the chain mediation hypothesis, indicating that P-O fit enhances satisfaction by first boosting self-efficacy, which in turn facilitates the development of perceived teacher competence.

The path via Teaching Self-Efficacy (β = 0.189) was significantly stronger than the path via Perceived Teacher Competence (β = 0.040). This suggests that self-efficacy is a more proximal and potent mediator than skill acquisition in the early stages of an internship. In summary, the total indirect effect (β = 0.298) accounted for 58.89% of the total effect (β = 0.506), indicating that the psychological mechanisms (efficacy and competence) play a dominant role in translating organizational fit into teachers’ job satisfaction.

## Discussion

The primary objective of this study was to investigate how Person-Organization (P-O) fit influences pre-service preschool teachers’ job satisfaction during their internship, and to unravel the underlying psychological mechanisms. By integrating Social Cognitive Theory (SCT), the Job Demands-Resources (JD-R) model, and Conservation of Resources (COR) theory, we constructed and validated a multi-pathway mediation model. Our empirical results extend beyond static correlations, revealing the dynamic psychological sequences that translate organizational compatibility into sustained professional well-being.

### P-O fit as a “protective shield” in the internship transition

Consistent with the Theory of Work Adjustment [63], our study confirms that P-O fit is a strong positive predictor of teachers’ job satisfaction (H1). However, extending beyond general corporate settings, our findings underscore the unique criticality of P-O fit for pre-service teachers facing the severe “reality shock” of early childhood education. To fully appreciate this ‘protective shield,’ it is necessary to unpack P-O fit into its structural dimensions [14] within the specific context of ECE. First, regarding value congruence (supplementary fit), early childhood education inherently demands intensive emotional labor [64]. When interns share the kindergarten’s core educational philosophies—such as child-centered versus teacher-directed approaches—they experience significantly reduced cognitive dissonance. They are not forced to enact ‘surface acting’ to comply with values they fundamentally disagree with. This aligns with recent evidence [13,30] demonstrating that value alignment acts as a primary buffer against early-career burnout, conserving crucial emotional resources. Second, regarding needs-supplies matching (complementary fit), the internship represents a period of high resource depletion. When the organization provides practical supplies that meet the interns’ developmental needs—ranging from adequate pedagogical materials to constructive mentor feedback—it fulfills the implicit psychological contract. This dual fulfillment—emotional alignment and practical resourcing—explains why a high P-O fit is not merely a contextual background, but an active, primary buffer directly anchoring interns’ early job satisfaction.

### Teaching self-efficacy: The psychological engine

Our findings indicate that Teaching Self-Efficacy (TSE) serves as a primary mediator (H7a), accounting for 37.35% of the total effect. This finding broadens the application of the JD-R model in teacher education. While previous literature often treats personal resources as innate traits, our results empirically validate that TSE is a highly malleable resource strictly contingent on environmental fit [33].

The pronounced disparity in mediation effect sizes—where TSE accounts for a substantial 37.35% of the total effect compared to PTC’s modest 7.91%—merits deeper theoretical consideration. This 5-fold difference empirically highlights the unique psychological landscape of the pre-service internship. Drawing upon Ma et al.’s insights into the early-career transition [65], pedagogical novices are typically situated in a ‘survival stage.’ During this vulnerable window, the immediate determinant of professional well-being is not the flawless execution of complex instructional strategies (competence), but rather the subjective confidence to navigate unpredictable classroom dynamics and manage emotional stress (efficacy). A high-fit environment accelerates this psychological adaptation. By providing clear value alignment and necessary resources, the kindergarten effectively neutralizes the initial ‘novice anxiety.’ Consequently, as Bandura’s framework suggests [21,34], interns in highly compatible settings interpret mentorship not as punitive evaluation, but as constructive ‘social persuasion’, thereby rapidly solidifying the efficacy beliefs that are paramount for early-stage job satisfaction.

We argue that P-O fit creates a sense of “psychological safety.” Interns in fitting environments do not need to expend cognitive energy monitoring their behavior to “fit in” or managing interpersonal conflicts. Instead, they can redirect these cognitive resources toward mastering teaching tasks, thereby enhancing their efficacy beliefs. This explains why interns with high P-O fit view classroom challenges as “manageable” rather than “threatening” [35].

### Perceived teacher competence: From belief to mastery

Our results also confirmed that Perceived Teacher Competence partially mediates the relationship (H7b). Through the lens of Self-Determination Theory [43], the actual mastery of core pedagogical skills fulfills the basic psychological need for competence, transitioning interns conceptually from peripheral observers to core contributors. Building upon the psychological safety and freed-up cognitive resources discussed earlier, interns can wholly dedicate their efforts to practical skill acquisition [32], consolidating their initial enthusiasm into tangible, intrinsic satisfaction.

### The chain mediation: The “virtuous cycle” of professional growth

A key finding of this research is the validation of the sequential chain mediation (H7c). Accounting for 13.64% of the total effect, this pathway provides empirical support for the sequential internalization of resources proposed by the Conservation of Resources (COR) theory [47].

This finding directly addresses a critical gap in existing literature. While previous empirical studies frequently examined efficacy and competence as isolated or parallel predictors of teacher well-being [66], our chain model confirms a strictly sequential “Environment → Belief → Action → Affect” developmental logic. This sequential evidence aligns with and extends the assertions of Skaalvik [67], proving that an advantageous external environment (P-O Fit) first establishes an initial psychological reservoir (Self-Efficacy). This psychological resilience provides the behavioral persistence required to acquire human capital (Competence), which ultimately manifests as sustained job satisfaction. By explicitly validating this psychological-to-behavioral sequence during internship socialization, our findings offer a more sophisticated, multi-step understanding of pre-service teacher development than previously established in single-mediator models.

## Implications

### Theoretical contributions

This study makes two significant contributions to the literature on teacher education and organizational psychology.

First, it constructs and validates an integrated “Environment-Psychology-Competence” framework, responding to the call for multi-pathway mechanisms in teacher education research. Unlike previous studies that treated teaching self-efficacy and competence as parallel or isolated constructs, our chain mediation model reveals a strictly sequential relationship: “Belief Precedes Action”. We provide empirical evidence that self-efficacy is a prerequisite for professional skill acquisition (Competence) in the early career stage. This finding enriches the Job Demands-Resources (JD-R) model by demonstrating that person-organization fit acts as a “contextual resource” that activates “personal resources” (efficacy), which are then converted into “human capital” (competence).

Second, this research extends the application of Person-Organization (P-O) Fit Theory to the specific context of pre-service teacher internships. Existing literature has largely focused on in-service teachers, overlooking the unique “vulnerability” of interns during the “window period” of professional identity formation. Our results corroborate that for novices facing “reality shock”, P-O fit functions as a critical “protective shield”. It buffers against the high emotional labor of kindergarten work, suggesting that the perception of fit is more critical for retention intentions in the early stages than actual job characteristics.

High needs-supplies fit actively promotes satisfaction by fostering psychological growth (efficacy) and professional mastery (competence). This dual function explains why mere “placement” (existence of a job) is insufficient for sustainability; only a “fitting” placement triggers the motivational cycle required for retention.

### Practical implications

It is worth noting that the sample for this study is derived from Changsha Normal University, a typical institution in China that has evolved from a vocational school to a normal undergraduate university. Institutions of this type are the primary providers of the preschool education workforce in China. Therefore, the practical implications derived from this context hold significant reference value for similar institutions facing the “expansion vs. attrition” paradox in China’s ECE sector. The “expansion vs. attrition” paradox in China’s ECE sector necessitates a shift from quantity-based to quality-based internship management.

#### For higher education institutions: From “assignment” to “mutual selection”.

1. Optimization of the Matching Mechanism: Universities should abandon random allocation in favor of a “Mutual Selection” mechanism. Prior to internships, assessments based on P-O fit scales can help match students’ educational values with the organizational culture of potential kindergartens. This aligns with our finding that value congruence is a primary driver of efficacy.
2. Pre-internship “Psychological Inoculation”: Given that self-efficacy is the dominant mediator, curriculum design should incorporate situational simulations and case studies before the internship begins. Strengthening students’ “psychological immunity” (efficacy) can help them better interpret and manage the inevitable “reality shock”.

#### For kindergartens: From “labor force” to “developmental support”.

Progressive Task Design: Following our chain mediation logic (Fit→Efficacy→Competence), mentorship programs should adopt a step-by-step approach.

Phase 1 (Adaptation): Mentors should focus on social support and assigning “low-risk” tasks to build the intern’s Self-Efficacy first.
Phase 2 (Development): Only after efficacy is established should complex teaching responsibilities be introduced to develop Perceived Teacher Competence.

This strategy prevents the “capacity overload” and burnout that occur when interns are forced to demonstrate competence before they believe in their own abilities.

### Limitations and future research

Despite the rigorous design, several limitations should be acknowledged to guide future inquiry.

1. Causal Inference: This study employed a cross-sectional design. Although our chain mediation model is theoretically grounded in Bandura’s Social Cognitive Theory, we cannot strictly determine causality (e.g., does higher competence lead to better fit, or vice versa?). Future studies should utilize longitudinal tracking designs (measuring fit at entry, efficacy at mid-term, and satisfaction at exit) to empirically capture the dynamic evolution of these variables.
2. Although our theoretical framework is also grounded in the Theory of Work Adjustment (TWA), our cross-sectional design limited our focus to job satisfaction as the primary outcome. TWA comprehensively models both satisfaction and actual tenure [63]. Future longitudinal research is strongly recommended to incorporate the retention component of TWA more explicitly. Tracking this cohort of pre-service teachers’ post-graduation would provide valuable empirical insights into how early internship satisfaction translates into actual career tenure within the early childhood education sector.
3. Measurement Bias: The assessment of Perceived Teacher Competence relied on self-report measures. While common in organizational psychology, self-reports may be subject to social desirability bias or the Dunning-Kruger effect. Future research would benefit from incorporating multi-source data, such as mentor evaluations or objective classroom observation scores, to enhance validity.

## Conclusion

The sustainability of the early childhood education workforce depends on the successful transition of pre-service teachers from “students” to “professionals”. This study addresses the critical issue of pre-service teacher attrition by validating a serial mediation model. We demonstrate that Person-Organization Fit is the cornerstone of a successful internship. Crucially, we reveal the sequential mechanism where “Belief Precedes Action”: a supportive organizational environment first ignites Teaching Self-Efficacy, which drives the acquisition of Perceived Teacher Competence, ultimately leading to Teachers’ Job Satisfaction.

These findings advocate for a highlight the need for a shift in teacher education—from a focus solely on “skill training” to a holistic approach that prioritizes “value alignment” and “self-efficacy”. By optimizing the fit between interns and kindergartens, stakeholders can effectively convert the “expansion of graduates” into a “stable workforce,” thereby ensuring the long-term sustainability of the ECE sector.

## Supporting information

S1 Data Dataset. Raw data of the questionnaire.
(XLS)

S1 File Scale English-Chinese correspondence. (ZIP)
