# Supplementary material for: Influence of person-organization fit on job satisfaction among pre-service preschool teachers of China: Mediation of teaching self-efficacy and perceived teacher competence
Source: PLoS One. 2026 Jun 8;21(6):e0351149. doi: 10.1371/journal.pone.0351149 (PMC13245781; doi:10.1371/journal.pone.0351149)
Supplement: S1 File — (ZIP) [file pone.0351149.s002.zip › Scale English-Chinese Correspondence/Scale English-Chinese Correspondence.docx]

**S1 Table.** Scale English- Chinese Correspondence

| **English** | **Chinese** |
| --- | --- |
| *Person–Organization (PO)* (Cable & DeRue, 2002; Wang Y. & Li, 2023) |  |
| My personal values are very similar to those of my kindergarten. | 我个人的价值观和我所在的幼儿园的价值观非常相似。 |
| My values and traits can be reflected in the internship at the kindergarten. | 我的价值观和特质能在幼儿园中得以体现。 |
| The material and spiritual resources provided by kindergarten work are very consistent with the job I want to find. | 幼儿园工作提供给我的物质和精神资源，和我想找的工作十分契合。 |
| The work of a kindergarten teacher is very much in line with my aspirations. | 幼儿教师的工作十分符合我的追求。 |
| The education and training I received are consistent with the needs of working in a kindergarten. | 我接受的教育及培训与幼儿园工作需要相匹配。 |
| *Teaching Self-Efficacy (TSE)* (Jin, 2020; Liu, 2012) |  |
| When children have difficulty understanding, I can provide some explanations of examples to help them understand. | 幼儿理解有困难时，我能提供一些解释或事例，帮助他们理解。 |
| I am able to use a variety of teaching methods to design activities that are lively and interesting. | 我能运用多种教学方法，将活动设计得生动有趣。 |
| I can easily handle the ‘disruptive’ behavior of some children during activities. | 我能轻而易举地处理活动中某些幼儿的“捣乱”行为。 |
| I can stimulate and mobilize children’s enthusiasm to participate in activities. | 我能激发、调动幼儿参与活动的积极性。 |
| During the activities, I can give children the opportunity to fully express and make decisions. | 活动中，我能给予幼儿充分表达和做决定的机会。 |
| I can stimulate children’s creativity and cultivate children’s creative thinking. | 我能激发幼儿的创造性，培养幼儿的创造性思维。 |
| *Perceived Teacher Competence (PTC)* (Yu, 2024) |  |
| I have mastered the basic methods of scientifically caring for young children in their daily lives. | 我掌握了科学照料幼儿日常生活的基本方法。 |
| I am familiar with the characteristics and laws of children’s physical and mental development. | 我熟知幼儿身心发展的特点和规律。 |
| I am able to assist the childcare staff in carrying out routine childcare and sanitation work in the class. | 我能够协助保育员开展班级常规保育和卫生工作。 |
| I can design educational activity plans based on children’s interests, needs, and age characteristics. | 我能够根据幼儿的兴趣需要和年龄特点设计教育活动方案。 |
| I am able to flexibly use a variety of methods to implement educational activities. | 我能够灵活运用多种方式实施教育活动。 |
| I can effectively observe children’s performance in activities and provide appropriate guidance when children need it. | 我能够有效观察幼儿在活动中的表现，在幼儿需要时给予适宜的指导。 |
| I can use observation, interviews, home-based cooperation and other methods to understand and evaluate young children. | 我能够运用观察、访谈、家园合作等多种方法，了解和评价幼儿。 |
| I can use evaluation results to analyze and improve educational activities. | 我能够运用评价结果，分析和改进教育活动。 |
| *Teachers' Job Satisfaction (TJS)* (Lu, 2024; Zhang, 2023) |  |
| I am contented with the teaching conditions of the kindergarten. | 我对幼儿园的教学条件感到满意。 |
| I am contented with the director’s management style. | 我对园长的管理方式感到满意。 |
| My efforts can be recognized by others. | 我的付出能得到他人的肯定。 |
| I find teaching interesting, and I am happy being around children. | 我觉得教学工作很有趣，和孩子们在一起很开心。 |
| My efforts receive positive responses from the children, and I enjoy the process. | 我的付出能够得到孩子们的积极回应， 我很享受这样的过程。 |

Note: PO = Person-Organization Fit; TSE = Teaching Self-Efficacy; PTC = Perceived teacher competence; TJS = Teachers’ Job Satisfaction.
